# Regulation of endothelial function by coagulation proteases in sepsis

**DOI:** 10.1186/cc9671

**Published:** 2011-03-11

**Authors:** JN McLaughlin, R Ramachandran, AM Kaynar, SD Shapiro, DC Angus, AB Malik

**Affiliations:** 1 University of Pittsburgh School of Medicine, Pittsburgh, PA, USA; 2 University of Illinois at Chicago, IL, USA

## Introduction

Thrombin and activated protein C (aPC) are two pleiotropic proteases whose opposing functions in hemostasis and endothelial function are dysregulated during sepsis. Exogenous supplementation of aPC, the ligand for endothelial protein C receptor (EPCR), is the only known therapeutic shown to reduce mortality in severe septic patients. Paradoxically, both thrombin and aPC signal the endothelium via the same receptor, protease-activated receptor-1 (PAR-1), by cleaving its N-terminus to produce an identical tethered ligand, yet result in opposing signaling networks. Once activated, PAR-1 triggers at least three separate signaling pathways (Gi, Gq, G13) and it is the relative contribution of each pathway that determines the endothelial response. Thrombin is a potent proinflammatory, endothelial barrier disruptive agonist, while aPC induces an anti-inflammatory and barrier protective phenotype, thought to be important to its therapeutic mechanism. We hypothesized that when bound to its ligand, aPC, EPCR functionally dimerizes with activated PAR-1, thereby altering its specificity for Gq, an important mediator of proinflammatory pathways in endothelial cells.

## Methods

We used bioluminescent resonance energy transfer to dynamically monitor the interaction of recombinant PAR-1 and EPCR in HEK cells. The effect of EPCR on PAR-1 G-protein selectivity was determined by EPCR siRNA knock down in cultured endothelial cells. Relative activation of Gq was determined by assaying agonist-induced intracellular calcium mobilization. G13 activation was determined by monitoring agonist-induced changes transendothelial electrical resistance across monolayers.

## Results

We found that in the absence of protease ligands, unactivated PAR-1 dimerizes with EPCR. However, proteolytically activated PAR-1/EPCR interaction was maintained with aPC but not thrombin. Both aPC and thrombin induced G13 signaling; however, aPC failed to activate Gq compared with thrombin. aPC-induced PAR-1/Gq signaling appears to be impaired by aPC-bound EPCR and is relieved when EPCR is depleted using siRNA.

## Conclusions

aPC-bound EPCR neutralizes the proinflammatory function of PAR-1 signaling by maintaining interaction with activated PAR-1, thereby abrogating Gq signaling. Thus it is not the difference in protease activation between thrombin and aPC, but rather the ability of aPC to direct PAR-1/EPCR dimerization that controls PAR-1 signaling, and thereby provides the therapeutic barrier protective/anti-inflammatory effects associated with aPC treatment.

